# Unlocking community capabilities for addressing social norms/practices: behavioural change intervention study to improve birth preparedness and complication readiness among pregnant women in rural Nigeria

**DOI:** 10.1186/s12884-020-03061-0

**Published:** 2020-06-22

**Authors:** Irene Ifeyinwa Eze, Chinyere Ojiugo Mbachu, Edmund Ndudi Ossai, Celestina Adaeze Nweze, Chigozie Jesse Uneke

**Affiliations:** 1 Department of Community Medicine, College of Medicine, Ebonyi State University, Abakaliki, Nigeria; 2 African Institute for Health Policy and Health Systems, Ebonyi State University, Abakaliki, Nigeria; 3 Department of Community Medicine, College of Medicine, University of Nigeria Enugu-Campus, Enugu, Nigeria

**Keywords:** Birth preparedness, Complication readiness, Pregnant women, Rural, Community, Nigeria

## Abstract

**Background:**

Maternal mortality is attributed to combination of contextual factors that cause delay in seeking care, leading to poor utilization of skilled health services. Community participation is one of the acknowledged strategies to improve health services utilization amongst the poor and rural communities. The study aimed at assessing the potentials of improving birth preparedness and complication readiness (BP/CR) using community-driven behavioural change intervention among pregnant women in rural Nigeria.

**Methods:**

A pre-post intervention study was conducted from June 2018 to October 2019 on 158 pregnant women selected through multi-stage sampling technique from 10 villages. Data on knowledge and practices of birth preparedness and utilization of facility health services were collected through interviewer-administered pre-tested structured questionnaire. Behavioural change intervention comprising of stakeholders’ engagement, health education, facilitation of emergency transport and fund saving system, and distribution of educational leaflets/posters were delivered by twenty trained volunteer community health workers. The intervention activities focused on sensitization on danger signs of pregnancy, birth preparedness and complication readiness practices and emergency response. Means, standard deviations, and percentages were calculated for descriptive statistics; and T-test and Chi square statistical tests were carried out to determine associations between variables. Statistical significance was set at *p*-value < 0.05.

**Results:**

The result showed that after the intervention, mean knowledge score of danger signs of pregnancy increased by 0.37 from baseline value of 3.94 (*p* < 0.001), and BP/CR elements increased by 0.27 from baseline value of 4.00 (p < 0.001). Mean score for BP/CR practices increased significantly by 0.22 for saving money. The proportion that had antenatal care (76.6%) and had facility delivery (60.0%) increased significantly by 8.2 and 8.3% respectively. Participation in Community-related BP/CR activities increased by 11.6% (*p* = 0.012).

**Conclusion:**

With the improvements recorded in the community-participatory intervention, birth preparedness and complication readiness should be promoted through community, household and male-partner inclusive strategies. Further evaluation will be required to ascertain the sustainability and impact of the programme.

## Background

Maternal mortality has become a public health concern worldwide due to persisting high rates in low-middle income countries including Nigeria [1, 2] The high prevalence of maternal mortality has been attributed to direct obstetric and indirect causes [1, 3]. The direct causes which include hemorrhage, pregnancy-related hypertensive disorders, puerperal infections, obstructed labour, and septic abortions complications accounts for more than 70% of maternal deaths worldwide. Indirect causes, such as pre-existing conditions including human immunodeficiency virus/ acquired immunodeficiency syndrome (HIV/AIDS), malaria, anemia, cardiovascular diseases and diabetes, contribute to more than 28% of maternal deaths [2, 4]. Poor knowledge of these danger signs and birth preparedness practices have been reported as contributory factors to maternal death [3, 4]. Maternal death has also been linked to combination of contextual factors: - socioeconomic, cultural and health system factors which cause delay in seeking care and ultimately lead to low utilization of skilled care [2].

Birth preparedness and complication readiness (BP/CR) has been advocated as a strategy to overcome the costly delays in decision-making to seek skilled services [5]. Birth preparedness and complication readiness is a process of planning for birth and anticipating actions to take in case of obstetric complications [6]. It is an essential part of antenatal care package in clinical setting, and a typical BP/CR plan would contain following elements: desired place of birth, preferred birth attendant, location of the closest facility for birth and in case of complications, funds for any expenses, supplies and materials to bring to the facility, an identified labour and birth companion, an identified support person to look after other children at home, identified transport to a facility for birth or in case of complications, and identification of compatible blood donors if needed [7]. In order to make the family ready for childbirth and any obstetric emergency, the concept of birth preparedness and complication readiness (BP/CR) is advocated to be introduced to the communities [1]. This would equip women and family members including husbands/partners to recognize danger signs, make birth preparedness arrangements, anticipate potential causes of delay in seeking care, and ensure timely use of skilled care [6, 8, 9]. Birth preparedness and Complication readiness can be accomplished through intervention such as Home Based Life Saving Skills (HBLSS) programme [9]. Such programme equips immediate family members including husbands/partners with knowledge to recognise danger signs, make birth preparedness arrangements and promote health-seeking behaviour. A review of impact of programmatic elements of BP/CR using diverse implementation strategies including community or home-based services reported increase in knowledge and birth preparedness practices, institutional deliveries and reduction of maternal and morbidity mortality [10–12].

One strategy to improve health services utilization amongst the poor and rural communities is to incorporate community participation into maternal programmes [13]. This would allow women and their family members to anticipate potential delays and ensure timely use of skilled care for birth and complications [6, 8]. The introduction of Focused Antenatal Care (ANC) was expected to increase client-provider contact time, allowing every woman to receive adequate individual counseling on the danger signs, birth preparation and emergency readiness and general maternal care. Despite this effort, studies have shown that at antenatal Care visits, the duration of contact between the health worker and the pregnant woman is low [14, 15]. More worrisome is the distribution of health manpower which is skewed towards urban populations with insufficient health workers at the primary health centres (PHCs) to serve the rural areas [16]. Currently, world health organization (WHO) recommend the use of community health workers in maternal and child health care following promising results in achieving reductions in neonatal mortality in low-income countries where such services have been implemented [10]. Applying innovative approach of using trained community health workers who are trusted members of the community will not only help in reducing the work load for the clinic staff but will provide opportunity for more contact time and easier delivery of BP/CR messages.

Many women in developing countries still give birth at home following traditional belief and custom [17, 18]. This is more so as pregnancy and childbirth are regarded as normal life events that do not require professional help [17, 18]. Ebonyi state has a total fertility rate of 5.3; and among women aged 15–49 years, 1 in 10 of them is currently pregnant [19]. Despite the high fertility rate, access to skilled care is yet not optimal. Nigeria demographic health survey reported that although the proportion that obtained antenatal care from skilled provider was 70.3%; delivery in health facility was 56.6% [20]. Low attendance to skilled care at birth has been associated with high maternal mortality. In Nigeria, pregnancy-related maternal mortality ratio for the 2018 National Demographic Health Survey (NDHS) is 556 deaths per 100,000 live births [20]; hence the need to address the problems linked to this high prevalence.

Poor knowledge of danger signs and emergency readiness among women have been reported in many studies as contributory to maternal mortality [21, 22]. According to the stage theory of behaviour change, individuals pass through series of stages including ‘pre-contemplation’ and ‘contemplation’ (recognizing the problem and assessing the ‘pros’ and ‘cons’ of the intended change) before making preparation for actual action [23]. As a result, many behavioural change interventions rely on health knowledge as a major awareness raising tool [24]. In the context of birth preparedness, awareness of the negative consequences of danger signs of pregnancy and childbirth has the potential to increase mothers’ (and family members) preparedness for birth and utilisation of skilled care. This study assessed the effect of community-driven behavioural change intervention on birth preparedness and complication readiness among pregnant women in rural Nigeria.

## Methods

### Description of study setting

The study was carried out in Ebonyi state, south eastern Nigeria, with a population of 2,176,947 [25]. Ebonyi state has thirteen local government areas (LGA) and a total 554 health facilities which render tertiary, secondary and primary health care services [16]. There is a strong presence of private hospitals in the state with about 60% of health services provided by mission hospitals [16]. The study site is Igbeagu community; one of the ancestral communities in Izzi LGA comprising of three communities/zones, five political wards and 58 villages. A mission hospital and two functional PHCs are located in the community and provide 24-h services including maternal care. Considerable proportion of the people in Izzi do not have formal education and major occupations of the inhabitants include farming, trading and crafts making. They have both monogamous and polygamous family setting and belong to several trans-generational associations and religious association.

#### Study design

A pre-post intervention study [26] was conducted in three phases: pre-intervention, intervention and post intervention. The first phase was a baseline survey using quantitative research method. The second phase involved instituting community-participatory behavioural change intervention. The third phase was post-intervention survey using the same questionnaires administered at baseline.

#### Study population

The study population consisted of pregnant women from the rural communities. Eligible participants were pregnant women, adults (aged 18 years and above) and permanent residents of the selected rural communities. Permanent residents were defined as people who had lived a minimum of 3 years in the selected communities. Those that declined consent to participate or unfit due to severe medical condition/impairment were excluded from the study.

### Sample size calculation and sampling technique

Using the formula for comparing two proportions [26] sample size of 158 participants was calculated with 11.5% as the change in proportion of subjects with health facility delivery [27] and power of 80%, after adjusting for 10% loss to attrition and design effect.

The study site-Izzi was purposively selected as a rural LGA and among the three LGAs in the state with the highest maternal mortality [16]. Igbeagu community was chosen because it had functional health facilities for effective delivery of 24-h health services. Multi-stage sampling technique (3-staged) was used in selecting the study participants. At the first stage, 2 wards were selected by simple random sampling from the 5 political wards in the community. At the second stage, 10 out of the 58 villages were selected by balloting. At the third stage, modified cluster sampling was used to recruit eligible participants from a cluster; (a cluster was defined as an autonomous village-: a locality governed by an appointed or elected traditional ruler/head). Mapping of all households with pregnant women in the selected clusters/villages was carried out. With equal allocation to each cluster, all eligible pregnant women were invited to participate in the study until the desired sample size was reached.

### Data collection

Prior to data collection, advocacy visits were paid to community leaders to solicit their support. A total of 10 research assistants (including 2 supervisors) from the community were trained to administer the questionnaire and the study instrument was pre-tested and back translated to ensure content and construct validity. The questionnaire was pre-tested on 15 randomly selected pregnant women in another community.

Data was collected at baseline and 6 months post-intervention using a structured interviewer-administered questionnaire (5-point Likert scale type) adapted from JHPIEGO training document [6]. Information was collected on knowledge and practices of BP/CR and participation in community BP/CR activities. The questionnaires were administered to the respondents at their homes and at a convenient time for participants. Each interview lasted about 45 min.

Post-intervention data was used to determine the effect of the intervention on knowledge and practice of birth preparedness and complication readiness, and participation in community BP/CR activities among participants.

### Description of intervention

A community-participatory behavioral change intervention was carried out after the baseline data collection. The intervention consisted of**:** (i) advocacy visits and stakeholder engagement for community buy-in, support, sustainability and ownership of the programme; (ii) training of volunteer community health workers (CHWs) on BP/CR; (iii) training of the household members on BP/CR; (iv) facilitation of Emergency Fund Saving Scheme (EFSS) and Emergency Transport Scheme (ETS) and training on BP/CR for the leaders of community associations/groups; and (v) production and distribution of posters/almanacs carrying messages on danger signs and BP/CR elements to participants.

Advocacy visits were made to community leaders to secure support and buy-in of the birth preparedness programme as well as promote sustainability and ownership of the programme. The principal researcher visited the traditional ruler and cabinet members; village heads; and leaders of women and transport workers’ association. This was followed by one-day training of 20 CHWs (10 males and 10 females). This was facilitated by the principal researcher using lectures/modules adapted from HBLSS training manual [28] and modified to fit the study context. The training was delivered through didactic lectures, pictorials, posters and discussion sessions. It consisted of 4 lecture topics covering danger signs of pregnancy, elements of BP/CR including emergency fund saving and transport schemes, promotion of early and complete ANC visits and utilization of health facility for skilled services. Each lecture lasted for 60 min (40 min of didactic and 20 min of questions and answers). Following the training, CHWs went round the community providing health education on BP/CR to pregnant women and their family members (husband/partner, children, and parents in law) in their homes and at convenient times. Pregnant women were also encouraged to identify and participate in BP/CR-related community activities such as group health fund saving and emergency transport scheme. Each household health education session lasted an average of 60 min. Participants were pre-informed of the programme through awareness campaigns provided by village heads. The language of communication was Izzi dialect.

In addition to house-to-house health education, CHWs distributed information education and communication (IEC) materials/handbills and posters to households and community leaders. The posters and handbills contained information on danger signs of pregnancy, child birth and after birth; elements of BP/CR and actions to be taken to prepare for birth. The purpose of the IEC materials was to visually reinforce the information communicated during lectures. The IEC materials also contained calendar dates and local market days to enable participants’ recall of clinic appointments.

Facilitation and strengthening of community support mechanisms for birth preparedness and complication readiness; −Emergency Fund Saving Scheme (EFSS) and Emergency Transport Schemes (ETS) were implemented. The Emergency Response Schemes were facilitated by the researcher and the trained community health workers and was targeted at leaders of community associations (men and women) and road transport workers. Lectures which focused on birth preparedness and complication readiness; importance of community emergency fund saving and transport schemes and the roles/responsibilities of community members in promoting birth preparedness were delivered. For emergency fund saving scheme, community groups/associations were sensitized on the need to save money for health emergencies and the mechanism and practice of effective saving. Members of the association were encouraged to make individual ‘health savings’ aside the monthly contribution/thrifts based on their capabilities. The health savings were to be collated centrally by officers designated for the role and saved in the association’s common purse. The health savings is to be disbursed to the contributors, interest free, on demand in health emergencies. For emergency transport scheme, members of the road transport workers were sensitized on the importance of the scheme in birth preparedness and prevention of pregnancy complications; and were also trained on prompt and safe transport services for maternal care. Volunteers in the scheme were expected to render services at subsidized fares. The transporters were to be incentivized through community recognitions; and the transport workers’ association were expected to permit the volunteers to take immediate turn of business transaction after an emergency service has been rendered.

#### Data management/analysis

The quality of the data was ensured by using trained research assistant and reviewing all questionnaires at the end of each day by the supervisors and principal researcher. Statistical Package for Social Sciences (IBM-SPSS) for Microsoft Window version 20 software was used for the data analysis. Frequency tables and bar charts were used to present the descriptive statistics and relevant means, standard deviations, and proportions were calculated. Likert scale analysis was based on mean rating (MNR) with a critical MNR of ‘3.0’ as the logical neutral point [29]. MNR of 3.0 and above implies good/high outcomes while MNR below 3.0 implies poor/low outcomes. Consequently, values ranging from 1.00–2.99 points were considered poor knowledge/practice whereas values ranging from 3.00–5.00 points were considered good knowledge/practice. T-test and Chi square tests were carried out to test for observed associations between variables. Statistical significance was set at *p*-value < 0.05.

## Results

A total of 158 pregnant women participated in the study at the beginning of the study. However, five participants were unavailable at post intervention stage giving loss to follow up/attrition rate of 3.2%. Most of the participants were in the 20–24 years’ age group (36.7%). Majority of the participants (92.4%.) were married, more than half (58.2%) had primary education and they are predominantly Christians (catholic-87.3%.). Common occupation of the participant was trading; followed by artisan and farming.

### Knowledge of danger signs and birth preparedness/complication readiness, and source of information

Table 1 shows that there was statistically significant increase in the discreet variables and sub-total mean knowledge scores of danger signs during pregnancy, at birth and after birth at the post-intervention stage. The overall mean knowledge score for danger signs increased significantly from the baseline mean score of 3.94 with mean increase of 0.37 (p = < 0.001).

**Table 1:** Mean knowledge score of danger signs among pregnant women in rural Ebonyi community

Variables	Pre-intervention Mean (SD); ***n*** = 158	Post-intervention Mean (SD); ***n*** = 153	Mean difference (***p***-value)**
**Danger sign during pregnancy**			
Vaginal bleeding	4.17 (0.85)	4.41 (0.55)	0.24 (0.003)*
Severe headache	4.29 (0.70)	4.42 (0.51)	0.13 (0.055)
Blurred vision	3.95 (0.92)	4.36 (0.61)	0.41(< 0.001)*
Convulsions	3.58 (1.16)	4.18 (0.81)	0.60(< 0.001)*
Swollen hands/face	4.25 (0.76)	4.50 (0.50)	0.25 (0.001)*
High fever	4.09 (0.83)	4.41 (0.60)	0.31(< 0.001)*
Loss of consciousness	3.84 (0.98)	4.24 (0.80)	0.40(< 0.001)*
Difficulty breathing	3.72 (1.09)	4.19 (0.83)	0.46(< 0.001)*
Severe weakness	4.10 (0.83)	4.40 (0.55)	0.29(< 0.001)*
Severe abdominal pain	4.03 (0.89)	4.37 (0.61)	0.33(< 0.001)*
Water breaks without labour	3.68 (1.06)	4.06 (0.96)	0.42(< 0.001)*
Increased/Reduced fetal movement	3.82 (4.25)	4.25 (0.76)	0.38 (0.001)*
Sub-total mean	3.96 (0.75)	4.31 (0.58)	0.35(< 0.001)*
**Danger sign during child birth**			
Severe vaginal bleeding	4.07 (0.93)	4.38 (0.62)	0.31(< 0.001)*
Severe headache	4.04 (0.96)	4.42 (0.55)	0.39(< 0.001)*
Convulsions	3.72 (1.09)	4.24 (0.93)	0.51(< 0.001)*
High fever	3.93 (0.96)	4.36 (0.65)	0.43(< 0.001)*
Loss of consciousness	3.73 (1.05)	4.22 (0.81)	0.48(< 0.001)*
Labor lasting> 12 h	4.04 (0.89)	4.35 (0.51)	0.31(< 0.001)*
Placenta not delivered 30 min after baby	4.13 (0.83)	4.55 (0.51)	0.42(< 0.001)*
Sub-total mean	3.95 (0.78)	4.35 (0.56)	0.41(< 0.001)*
**Danger sign after child birth**			
Severe bleeding	4.20 (0.81)	4.45 (0.51)	0.25(< 0.001)*
Severe headache	4.18 (0.78)	4.43 (0.51)	0.25 (0.001)*
Convulsions	3.76 (0.96)	4.23 (0.73)	0.47 (0.001)*
High fever	3.99 (0.90)	4.33 (0.57)	0.35(< 0.001)*
Loss of consciousness	3.73 (1.04)	4.25 (0.69)	0.51(< 0.001)*
Malodorous Vaginal Discharge	3.76 (0.96)	4.20 (0.76)	0.44(< 0.001)*
Difficulty breathing	3.73 (1.04)	4.25 (0.75)	0.51(< 0.001)*
Severe weakness	4.11 (0.81	4.37 (0.54)	0.27(< 0.001)*
Sub-total mean	3.94 (0.77)	4.28 (0.58)	0.37(< 0.001)*
**Overall mean knowledge score**	3.94 (0.74)	4.32 (0.56)	0.37 (< 0.001)*

*statistical significance; **T-Test for statistical significance

Table 2 shows that the mean knowledge scores for BP/CR elements was increased significantly for most of the variables at post intervention. Also, there was significant increase in the overall mean knowledge scores for BP/CR elements from 4.0 at baseline to 4.27 at post-intervention (*p* < 0.001).

**Table 2:** Mean knowledge score of birth preparedness/complication readiness among pregnant women in rural Ebonyi community

Variables	Pre-intervention Mean (SD); n = 158	Post intervention Mean (SD); n = 153	Mean difference (***p***-value)**
**BP/CR elements**			
Identify mode of transport	3.94 (0.93)	4.32 (0.55)	0.37 (< 0.001)*
Save Money (for emergency)	4.10 (0.82)	4.33 (0.55)	0.23 (0.005)*
Identify Blood Donor	3.66 (0.93)	4.16 (0.70)	0.50 (< 0.001)*
Identify Skilled Provider	3.89 (0.77)	4.19 (0.62)	0.30 (< 0.001)*
Identify where to go for emergency	4.04 (0.90)	4.27 (0.86)	0.23 (0.008)*
Identify where to go for birth	4.16 (0.86)	4.28 (0.61)	0.12 (0.150)
Prepare Birth Kit	4.22 (0.83)	4.35 (0.53)	0.14 (0.084)
**Total mean knowledge score**	4.00 (0.71)	4.27 (0.52)	0.27 (< 0.001)*

*statistical significance; **T-Test for statistical significance; BR/CR Birth preparedness and Complication readiness

**Fig. 1:**
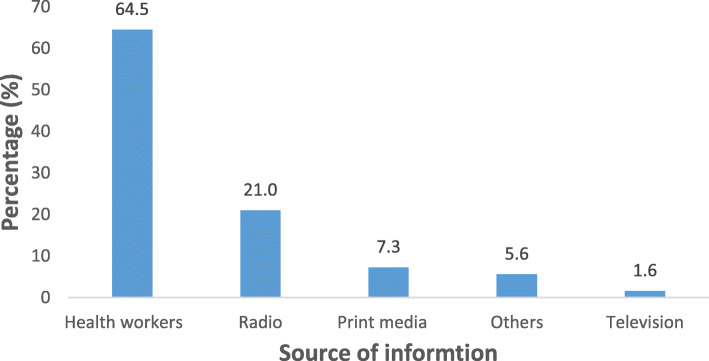
Source of Information on Birth Preparedness/Complication Readiness among Pregnant Women in Rural Ebonyi Community

Figure 1 shows that the main sources of information on danger signs and birth preparedness/complication readiness was health workers (64.5%), followed by radio (21.0%).

### Birth preparedness and complication readiness practices

Table 3 shows that the mean score increased for all the discreet birth preparedness variables. However, statistically significant increase was observed at post intervention stage only for the practice of saving money for birth (*p* = 0.004).

**Table 3:** Mean scores of Birth preparedness/complication readiness practices among pregnant women in rural Ebonyi community

Variables	Pre-intervention Mean (SD);***n*** = 94	Post-intervention Mean (SD); ***n*** = 127	Mean difference (***p***-value)**
**Adequacy of birth preparedness**			
Identify mode of transport	3.99 (1.26)	4.11 (1.21)	0.12 (0.471)
Save Money (for emergency)	4.47 (0.68)	4.69 (0.48)	0.22 (0.004)*
Identify Blood Donor	3.17 (1.55)	3.40 (1.40)	0.23 (0.228)
Identify Skilled Provider	3.68 (1.28)	3.83 (1.15)	0.15 (0.351)
Identify where to go for emergency/ birth	4.12 (1.26)	4.06 (1.15)	−0.06 (0.704)
Prepare Birth Kit	4.60 (0.87)	4.75 (0.82)	0.15 (0.184)
**Total mean score**	4.00 (0.81)	4.14 (0.69)	0.14 (0.169)

* Statistically significant; **T -Test for statistical significance

Table 4 shows there was significant increase in the proportion of pregnant women that participated in any birth preparedness-related community activities with a percentage increase of 11.6% at post intervention (*p* = 0.012). A statistically significant increase was noted in the proportion of pregnant women that participated in community activity of organizing/identifying transport service (*p* = 0.024).

**Table 4:** Participation in birth preparedness-related community activities by pregnant women in rural Ebonyi community

Variable	Pre-intervention Frequency(%) n = 158	Post intervention Frequency(%) n = 153	%Difference (***p***-value)**
Participated in any BP/CR- related community activity in the past 6 months			
Yes	24 (15.2)	41 (26.8)	11.6 (0.012)*
No	134 (84.8)	112 (73.2)	
**Activities participated**			
Transport service			
Yes	8 (19.5)	11 (45.8)	26.3 (0.024)*
No	16 (80.5)	30 (54.2)	
Ways to save money			
Yes	9 (37.5)	18 (43.9)	6.4 (0.613)
No	15 (62.5)	23 (56.1)	
Ways to get blood donated			
Yes	2 (8.3)	6 (14.6)	6.3 (0.445)
No	22 (91.7)	35 (85.4)	

* Statistically significant; **χ2-Test for statistical significance; BR/CR Birth preparedness and Complication readiness

### Utilization of health facility for skilled care and delivery

Table 5 shows statistically significant decrease in the experience of serious pregnancy related problems with percentage difference of 5.6% at post intervention stage (*p* = 0.018). There was statistically significant increase in the proportion that attended ANC and practiced facility delivery with percentage difference of 8.2% (*p* = 0.004) and 8.3% (*p* = 0.039) respectively. Although this was not statistically significant, there was increase in the proportion of pregnant women that sought assistance for serious pregnancy related problems from private hospitals.

**Table 5:** Utilization of health facility for skilled care and delivery among pregnant in rural community, Ebonyi State

Variable	Pre-intervention Frequency(%) n = 158	Post intervention Frequency(%) n = 153	%Difference (***p***-value)**
Experience of any serious health problems related to pregnancy			
Yes	37 (23.4)	20 (13.1)	5.55 (0.018)*
No	121 (76.6)	133 (86.9)	
Sought assistance for the problems			
Yes	29 (78.4)	19 (95.0)	2.69 (0.101)
No	8 (21.6)	1 (5.0)	
Where assistance was first sort			
Government. health centre	25 (86.2)	12 (63.1)	3.64 (0.161)
Private / mission hospital	3 (10.3)	6 (31.6)	
Traditional birth attendants (TBAs) / Others	1 (3.5)	1 (5.3)	
Attended ANC during this pregnancy			
Yes	121 (76.6)	136 (88.9)	8.20 (0.004)*
No	37 (23.4)	17 (11.1)	
Where you intend to give/gave birth to your child			
Respondents home	28 (29.5)	25 (20.1)	8.31 (0.039)*
TBA’s home	5 (5.3)	4 (3.2)	
Hospital/health centre/maternity home	57 (60.0)	94 (75.7)	
Others	5 (5.3)	1 (0.8)	

* Statically significant; **χ2-Test for statistical test**;** Others-Neighbors, Relatives, Friend

## Discussion

### Knowledge of danger signs and birth preparedness

This study reported good knowledge of danger signs and birth preparedness elements comparable with the finding in a study carried out in southern Nigeria, Edo State [5] and in Ghana [30] where good knowledge was reported. The high knowledge reported in this study could be explained by possible reliable information from health workers which was the main source of information. The result however, contradicts other studies conducted where women were shown to have insufficient knowledge of birth preparedness in Nigeria [31–33] and in other countries [34–36]. A community-based survey in northern Nigeria found that only 32.0% knew any critical danger sign relating to pregnancy and delivery [37], and less than half (44.6%) in Northeast Ethiopia [36].

After the intervention, this study found significant increase in knowledge of almost all the discreet variables as well as composite mean scores of danger signs of pregnancy and birth preparedness. This implies that rural women are potentially educable regarding maternal health notwithstanding the low educational level, as primary education was the highest level for most of the participants. Consistent with our study, the mean composite knowledge scores increased significantly in a pre-post intervention study on impact of an educational session on knowledge of safe motherhood in Benin city, Nigeria [38]. Another study in northern Nigeria reported significantly greater improvements in the intervention communities that received additional demand-side interventions, as about 22% of the women knew at least 4 maternal danger signs compared with 10% at baseline [39]. This finding also corroborates a participatory multi-sectoral intervention study in Uganda which reported that the intervention significantly increased the knowledge of at least three obstetric danger signs [40]. Other studies have shown that community-based intervention on BP/CR were effective in raising women’s knowledge of danger signs [9, 41, 42]. Such programmes should therefore be intensified and sustained as knowledge enhances cue for action.

### Practices of birth preparedness and complication readiness

This study found good birth preparedness practices among pregnant women with mean score of above 3.0 in all the variables. Also, However, functional items needed for birth (preparing birth kit), seemed to be given precedence over other birth preparedness practices as shown by the very high mean score. The good birth preparedness practices noted in this study corroborates the findings in assessment of BP/CR practices in Tanzania [43] and Uganda [44] where discrete actions (e.g. financial savings and identification of place to deliver) were taken by 75% of respondents. This finding is however, contrary to what was reported in some studies carried out among pregnant women in rural communities in Edo State [5] and in northern Nigeria [37, 45] where less than half of the respondents were well prepared. In this study, less than a third of the pregnant women participated in birth preparedness-related community activities. Similar low participation in community activities related to birth preparedness was reported in Ghana where less than 5% were involved in emergency transport arrangements and emergency financial support services [30]. One strategy to improve health services utilization amongst rural communities is to incorporate community participation into maternal health programmes [13]. There is therefore need for pregnant women to be aware of this gain and hence be sensitized to participate in birth preparedness-related community activities.

The proportion that had antenatal care (76.6%) and health facility delivery practices (60%) compares with other studies carried out in Ghana [30] and Tanzania [43]. The lower proportion in practice of facility delivery noted in this study could be explained by presence of some barriers common in rural areas such as inaccessibility in terms of distance, transport and cost [46]. At variance with our findings, low utilization of skilled care was reported among women surveyed in in Northern Nigeria where only 26% had any antenatal care and only 13% delivered in a facility with a skilled birth attendant [37]. Another study in South West Shoa Zone, Ethiopia reported use of skilled birth attendant in only 28.6% of the respondents [46]. Birth preparedness and complication readiness is a strategy based on the theory that preparing for childbirth and being ready for complications will promote timely use of skilled maternal care. These findings highlight the importance of focusing on all aspect of birth preparation including attitude, involvement in decision making, emergencies preparedness and community participation in interventions aimed at increasing women’s use of skilled maternity care.

After the intervention, our study showed significant increase in some birth preparedness practices like saving money, attending ANC visit and facility delivery practices. Experience of serious health problem related to pregnancy decreased significantly at post intervention and a significant increase was noted in the proportion that participated in community BP/CR related activities. The improvement in birth preparedness practices observed among the participants could be attributed to the good knowledge of birth preparedness noted in this study. Also, the improvement in skilled care utilization observed in this study can be explained by the diverse BCC strategies used. Consistent with our finding, some behavioural change community-based intervention studies reported improvement in preparedness for childbirth and increased deliveries at health facilities in rural communities [41, 42]. Behavioural Change Communication (BCC) has been viewed as effective strategy for improving access to maternal health services [41]. An exploratory study of community perceptions of behaviour change communication interventions of the maternal neonatal and child health programme in rural Bangladesh reported that community-based BCC interventions are well accepted by the community people [47]. Inter-personal communication (IPC) is considered an essential aspect of everyday life and community members probably appreciated the personal interaction with their community members that served as community health workers. Birth preparedness using community-based BCC programmes should therefore be encouraged.

#### Limitations

The data collected on birth preparedness practices relied on self-report and recall from participants and this is subject to reporting/response bias. There is strong belief that practices such as health facility delivery and birth preparedness are favourable behaviours, and respondents may prefer to provide socially desirable responses rather than the truth. This may lead to responding more positively to these practices than actually occurred, making over reporting a possibility. The CHWs minimized this by building rapport and trust since they are fellow community members. The study design was a pre and post intervention without control which may limit the interpretation of the observed change resulting solely from the intervention as there may be other confounders. The duration allowed after the intervention may have influenced the extent of observed change as most community activities may still be at preliminary stages. Hence the need for long term reassessment of impact and sustainability of the progromme.

## Conclusions

This study highlighted that the majority of pregnant women had good knowledge of danger signs of pregnancy and birth preparedness but seemed to place importance on functional items needed for delivery rather than on arranging transport or identifying a skilled care provider, blood donor or health facility. This emphasizes the need for emergency preparedness to women during sensitization on birth preparedness. As shown in this study, community participation can be effective as a mechanism for addressing shortage of skilled manpower for safe motherhood especially in rural areas. Hence, there is need for multi-stakeholder involvement-; involving not only women, but also men, family members, communities, and health care providers in birth preparedness and complication readiness programmes.

## Data Availability

The dataset used for this study is readily available and can be obtained from the corresponding author on reasonable request.

## Abbreviations

ANC: Antenatal care
BCC: Behavioural Change Communication
BP/CR: Birth Preparedness and Complication Readiness
CHWs: Community health workers
EBSU: Ebonyi State university
EFSS): Emergency Fund Saving Scheme
ETS: Emergency Transport Scheme
HBLSS: Home Based Life Saving Skills
HIV/AIDS: Human Immunodeficiency virus/ Acquired immunodeficiency syndrome
IEC: Information Education and Communication
IPC: Inter-personal communication
LGA: Local Government Areas
MNR: Mean rating
PHCs: Primary health centres
REC: Research and Ethical Committee
SPSS: Statistical Package for Social Sciences
TBAs: Traditional Birth Attendants
UREC: University Research Ethical Committee
WHO: World Health Organization

## References

[1] Bank TW, The World Bank (2010). Trends in Maternal Mortality : 1990 to 2010. Organization.

[2] Alkema L, Chou D, Hogan D, Zhang S, Moller A, Gemmill A (2015). Global , regional , and national levels and trends in maternal mortality between 1990 and 2015 , with scenario-based projections to 2030 : a systematic analysis by the UN Maternal Mortality Estimation Inter-Agency Group. Lancet.

[3] Say L, Chou D, Gemmill A, Tunçalp Ö, Moller A-B, Daniels J (2014). Global causes of maternal death: a WHO systematic analysis. Lancet.

[4] Bongaarts J (2016). WHO, UNICEF, UNFPA, World Bank Group, and United Nations population division trends in maternal mortality: 1990 to 2015 Geneva: World Health Organization, 2015. Popul Dev Rev.

[5] Ibadin SH, Adam VY, Adeleye O, Okojie OH (2016). Birth preparedness and complication readiness among pregnant women in a rural community in southern Nigeria. S Afr J Obstet Gynaecol.

[6] Jhpiego (2004). Monitoring birth preparedness and complication readiness: tools and indicators for maternal and newborn health. Balt JHPIEGO.

[7] WHO. Birth and emergency preparedness in antenatal care. Intergrated Manag Pregnancy Childbirth. 2006;6 Available from: http://scholar.google.com/scholar?hl=en&btnG=Search&q=intitle:Birth+and+emergency+preparedness+in+ antenatal+care#3.

[8] Darmstadt GL, Choi Y, Arifeen SE, Bari S, Rahman SM, Mannan I (2010). Evaluation of a cluster-randomized controlled trial of a package of community-based maternal and newborn interventions in Mirzapur, Bangladesh. PLoS One.

[9] Miltenburg AS, Roggeveen Y, Shields L, van Eltere M, van Roosmalen J, Jelle Stekelenburg AP, Roggeveen Y, Shields L, Van M (2015). Impact of birth preparedness and complication readiness interventions on birth with a skilled attendant : a systematic review. PLoS One.

[10] Bhutta ZA, Lassi ZS, Pariyo GHL. Global experience of community health workers for delivery of health related Millennium Development Goals: a systematic review, country case studies, and recommendations for integration into national health systems. Geneva World Heal Organ Glob Heal Work Alliance. 2010. p. 1–2. https://www.who.int/workforcealliance/knowledge/publications/alliance/Global_CHW_web.pdf.

[11] Lassi Z, Das J, Salam RBZ (2014). Evidence from community level inputs to improve quality of care for maternal and newborn health: interventions and findings. Reprod Heal.

[12] Lassi ZS, Bhutta ZA. Community-based intervention packages for reducing maternal and neonatal morbidity and mortality and improving neonatal outcomes. Cochrane Database Syst Rev. 2015;2015(3):1–79. 10.1002/14651858.CD007754.pub2. 10.1002/14651858.CD007754.pub3 PMC8498021 25803792

[13] Indriani D (2012). Community participation as a strategy in reducing maternal and child mortality in rural areas: A Literature review.

[14] Magoma M, Requejo J, Merialdi M, Campbell OM, Cousens S, Filippi V (2011). How much time is available for antenatal care consultations? Assessment of the quality of care in rural Tanzania. BMC Pregnancy Childbirth.

[15] Pembe AB, Lindmark G (2010). Quality of antenatal care in rural Tanzania : Counselling on pregnancy danger signs Quality of antenatal care in rural Tanzania : counselling on pregnancy danger signs. BMC Pregnancy Childbirth.

[16] Ebonyi State Ministry of health (2010). Ebonyi state government strategic health development plan. Abakaliki.

[17] Wild K, Barclay LKP (2010). Birth choices in TimorLeste: a framework for understanding the use of maternal health services in low resource settings. Soc Sci Med.

[18] Agus Y, Shigeko HPS (2012). Rural Indonesia women’s traditional beliefs about antenatal care. BMC Res Notes.

[19] National Population Commission. Nigeria Demographic and Health Survey 2013. Natl Popul Comm. 2014. p. 201–21. https://www.bing.com/search?q=National+Population+Commission.+Nigeria+Demographic+and+Health+Survey+2013.+Natl+Popul+Comm.+2014%3A+201-221&cvid=0d3527f33b264189b1d7a848511d6780&FORM=ANAB01&PC=U531.

[20] National Population Commission. Nigeria demographic and health survey 2018 Key Indicators Report. MD Natl Popul Comm ORC; 2019. p. 1–69. Available from: https://www.dhsprogram.com/pubs/pdf/PR118/PR118.pdf A https://www.scholar.google.com/scholar?hl=en&btnG=Search&q=intitle:Nigeria+Demographic+And+Health+Survey#0.

[21] Hailu M, Gebremariam A, Alemseged FDK (2011). Birth preparedness and complication readiness among pregnant women in southern Ethiopia. PLoS One.

[22] Kabakyenga JK, Ostergren PO, Turyakira EPK (2011). Knowledge of obstetric danger signs and birth preparedness practices among women in rural Uganda. Reprod Heal.

[23] Prochaska JOVW (1997). The transtheoretical model of health behavior change. Am J Health Promot.

[24] Aboud FESD (2012). Challengestochanging healthbehaviours in developing countries: a critical overview. Soc Sci Med.

[25] National Population Commission (2009). Legal Notice on Publication of 2006 Census Final Results. Fed Repub Niger Off Gazette.

[26] Onwuasaigwe C (2010). Sampling and sampling methods. Principles and Methods of Epidemiology.

[27] CIA World Factbook (2018). Nigeria Maternal mortality rate. index Mundi.

[28] Sibley L, Buffington ST, Haileyesus D (2004). The American College of Nurse-Midwives’ home-based lifesaving skills program: a review of the Ethiopia field test. J Midwifery Womens Heal.

[29] Uneke CJ, Ezeoha AE, Uro-Chukwu H, Ezeonu CT, Ogbu O, Onwe F, Edoga C (2015). Improving Nigerian health policymakers’ capacity to access and utilize policy relevant evidence: outcome of information and communication technology training workshop. Pan Afr Med J..

[30] Kuganab-lem RB, Dogudugu R, Kanton L. Birth Preparedness and Complication Readiness: A Study of Postpartum Women in a Rural District of Ghana. Scientific & Academic Publishing. 2015;4(6):225–33. Available from: 10.5923/j.phr.

[31] Barros AJ, Ronsmans C, Axelson H, Loaiza E, Bertoldi AD, Franca GV (2012). Equity in maternal, newborn, and child health interventions in countdown to 2015: a retrospective review of survey data from 54 countries. Lancet.

[32] Ogunmola OJ, Olaifa AO, Oladapo OO, Babatunde OA (2013). Prevalence of cardiovascular risk factors among adults without obvious cardiovascular disease in a rural community in Ekiti state, Southwest Nigeria. BMC Cardiovasc Disord.

[33] Shivalli S, Srivastava RK, Singh GP (2015). Trials of Improved Practices ( TIPs ) to Enhance the Dietary and Iron-Folate Intake during Pregnancy- A Quasi Experimental Study among Rural Pregnant Women of.

[34] Oiyemhonlan B, Udofia E, Punguyire D. Identifying obstetrical emergencies at Kintampo Municipal Hospital: a perspective from pregnant women and nursing midwives. Afr J Reprod Health. 2013;17(2):129–40 Available from: http://ovidsp.ovid.com/ovidweb.cgi?T=JS&PAGE=reference&D=medl&NEWS=N&AN=24069758. 24069758

[35] Markos D, Bogale D (2014). Birth preparedness and complication readiness among women of child bearing age group in Goba woreda, Oromia region, Ethiopia. BMC Pregnancy Childbirth.

[36] Wilunda C, Quaglio G, Putoto G, Takahashi R, Calia F, Abebe D, et al. Determinants of utilisation of antenatal care and skilled birth attendant at delivery in South West Shoa Zone , Ethiopia : a cross sectional study. Reprod Health. 2015:1–12. Available from:. 10.1186/s12978-015-0067-y. 10.1186/s12978-015-0067-y PMC4592558 26432298

[37] Doctor HV, Findley SE, Cometto GAG (2013). Awareness of critical danger signs of pregnancy and delivery, and preparations for delivery, and utilization of skilled birth attendants in Nigeria. J Heal Care Poor Underserved.

[38] Adeleye OA, Okonkwo CA (2016). Changes in Men’s Knowledge & Attitudes Following Health Education on their Role in Preventing Maternal Deaths: An Exploratory Survey in a Nigerian Community. Soc Med.

[39] Findley SE, Doctor HV, Ashir GM, Kana MA, Mani AS, Green C (2015). Reinvigorating health systems and community-based services to improve maternal health outcomes: case study from northern Nigeria. J Prim Care Community Health.

[40] Muhumuza R, Tetui M, Bua J, Ekirapa-kiracho E. Effect of a participatory multisectoral maternal and newborn intervention on birth preparedness and knowledge of maternal and newborn danger signs among women in Eastern Uganda : a quasi-experiment study. Glob Health Action. 2017;10(0) Available from:. 10.1080/16549716.2017.1362826. 10.1080/16549716.2017.1362826 PMC5645681 28849729

[41] August F. Effect of Home Based Life Saving Skills Education on Knowledge of Obstetric Danger Signs, Birth Preparedness, Utilization of Skilled Care & Male Involvement. Acta Univ Ups Uppsala. 2016;(ISSN 1651–6206):13–57 Available from: https://uu.diva-portal.org/smash/get/diva2:894992/FULLTEXT01.pdf.

[42] August F, Pembe AB, Mpembeni R, Axemo P, Darj E. Effectiveness of the Home Based Life Saving Skills training by community health workers on knowledge of danger signs , birth preparedness , complication readiness and facility delivery , among women in Rural Tanzania. BMC Pregnancy Childbirth. 2016:1–12. Available from:. 10.1186/s12884-016-0916-x. 10.1186/s12884-016-0916-x PMC4890507 27251052

[43] Tancred T, Marchant T, Hanson C, Schellenberg J, Manzi F. Birth preparedness and place of birth in Tandahimba district , Tanzania : what women prepare for birth , where they go to deliver , and why. BMC Pregnancy Childbirth. 2016:1–9. Available from:. 10.1186/s12884-016-0945-5. 10.1186/s12884-016-0945-5 PMC4947316 27422526

[44] Bitew Y, Awoke W, Chekol S (2016). Birth preparedness and complication readiness practice and associated factors among pregnant women, Northwest Ethiopia. Int Sch Res Not.

[45] Iliyasu Z, Abubakar IS, Galadanci HS, Aliyu MH (2010). Birth preparedness, complication readiness and fathers’ participation in maternity care in a northern Nigerian community. Afr J Reprod Health.

[46] Timsa L, Marrone G, Ekirapa E, Waiswa P. Strategies for helping families prepare for birth: experiences from eastern central Uganda. Global HealthAction. 2015;8(1):1–9. Available from: 10.3402/gha.v8.23969. 10.3402/gha.v8.23969 PMC4385208 25843492

[47] Rahman A, Leppard M, Rashid S, Jahan N, Nasreen HE (2016). Community perceptions of behaviour change communication interventions of the maternal neonatal and child health programme in rural Bangladesh: an exploratory study. BMC Health Serv Res.

